# Purification, characterization and immunogenicity assessment of glutaminase free L-asparaginase from *Streptomyces brollosae* NEAE-115

**DOI:** 10.1186/s40360-018-0242-1

**Published:** 2018-08-23

**Authors:** Noura El-Ahmady El-Naggar, Sahar F. Deraz, Sara M. El-Ewasy, Ghada M. Suddek

**Affiliations:** 1 Department of Bioprocess Development, Genetic Engineering and Biotechnology Research Institute, City of Scientific Research and Technological Applications, Alexandria, Egypt; 2 Department of Protein Research Genetic Engineering and Biotechnology Research Institute, City of Scientific Research and Technological Applications, Alexandria, Egypt; 3 Department of Pharmacology and Toxicology, Faculty of Pharmacy, Mansoura University, Mansoura, Egypt

**Keywords:** *Streptomyces brollosae* NEAE-115, L-asparaginase, Purification, DEAE Sepharose CL-6B, Characterization, Immunogenicity assessment

## Abstract

**Background:**

L-asparaginase is a potential therapeutic enzyme widely used in the chemotherapy protocols of pediatric and adult patients with acute lymphoblastic leukemia. However, its use has been limited by a high rate of hypersensitivity in the long-term used. Hence, there is a continuing need to search for other L-asparaginase sources capable of producing an enzyme with less adverse effects.

**Methods:**

Production of extracellular L-asparaginase by *Streptomyces brollosae* NEAE-115 was carried out using submerged fermentation. L-asparaginase was purified by ammonium sulphate precipitation and pure enzyme was reached using ion-exchange chromatography, followed by enzyme characterization. Anticancer activity towards Ehrlich Ascites Carcinoma (EAC) cells was investigated in female Swiss albino mice by determination of tumor size and the degree of tumor growth inhibition. The levels of anti-L-asparaginase IgG antibodies in mice sera were measured using ELISA method.

**Results:**

The purified L-asparaginase showed a total activity of 795.152 with specific activity of 76.671 U/mg protein and 7.835 − purification fold. The enzyme purity was confirmed by using SDS–PAGE separation which revealed only one distinctive band with a molecular weight of 67 KDa. The enzyme showed maximum activity at pH 8.5, optimum temperature of 37 °C, incubation time of 50 min and optimum substrate concentration of 7 mM. A Michaelis-Menten constant analysis showed a K_m_ value of 2.139 × 10^− 3^ M with L-asparagine as substrate and V_max_ of 152.6 UmL^− 1^ min^− 1^. The half-life time (T_1/2_) was 65.02 min at 50°С, while being 62.65 min at 60°С. Furthermore, mice treated with *Streptomyces brollosae* NEAE-115 L-asparaginase showed higher cytotoxic effect (79% tumor growth inhibition) when compared to commercial L-asparaginase group (67% tumor growth inhibition).

**Conclusions:**

The study reveals the excellent property of this enzyme which makes it highly valuable for development of chemotherapeutic drug.

## Background

L-asparaginase (EC 3.5.1.1) is the enzyme that catalyzes the hydrolysis of L-asparagine to L-aspartic acid and ammonia. The leukemic cells cannot synthesize L-asparagine due the absence of L-asparagine synthetase [1]. “These cells require huge amount of L-asparagine and rely on the exogenous sources for their proliferation and survival. Thus, intravenously injection of L-asparaginase in cancer patients destroys the exogenous L-asparagine and results in depletion of L-asparagine in the blood. L-asparagine starvation affecting selectively the tumor cells, since they are unable to complete protein synthesis [2]”. On the contrary, normal cells are protected from L-asparagine starvation due to their ability to synthesize this amino acid which is essential for both cell survival and protein synthesis [3]. Hence, in combination therapy of children acute lymphoblastic leukemia (ALL), L-asparaginase is considered as the effective drug of choice [4, 5]. In addition, L-asparaginase is significantly used for the treatment of other malignant disorders including lymphosarcoma, acute lymphoblastic leukemia, chronic lymphocytic leukemia, acute myelomonocytic leukemia, acute myelocytic leukemia, Hodgkin disease, melanosarcoma and reticulosarcoma [6].

Currently, two formulations of L-asparaginases are effectively in clinical use for ALL treatments, one from *Erwinia chrysanthemi* and the other from *E. coli.* However, its therapeutic long-term use leads to unpleasant side effects as a result of exerting normal cells toxicity, patient’s hypersensitivity and the rapid clearance of the enzyme from the blood stream which limited its administration. Clinical uses indicate that the L-asparaginases toxicity is mainly resulted from enzyme glutaminase activity and bacterial endotoxins in enzyme preparations [7]. Hepatotoxicity is the important adverse effect in the majority of patients [8], L-asparaginase causes a wide spectrum of side effects, such as skin rashes, fever, hepatic dysfunction, leucopoenia, pancreatitis, diabetes, neurological seizures and abnormal coagulation tests that may lead to intracranial thrombosis or haemorrhage [3]. L-asparaginase purification consider as an essential step for its physical and biological characterization. Moreover, for effective therapeutic administration with less adverse effects, L-asparaginase preparation must be free of any contaminants and impurities which also required efficient purification step.

In an attempt to minimize the allergenic reactions caused by impurities, enormous numbers of research groups have achieved production and purification of L-asparaginase [9]. Manivasagan et al. [10] reported that actinomycetes have been recognized to be a good source for L-asparaginase production, in particular *Streptomyces* species which are responsible for providing almost half of the useful drugs of bioactive secondary metabolites, especially antibiotics followed by enzymes and anti cancer agents. Several *Streptomyces* species have been explored for L-asparaginase production such as *Streptomyces parvus* NEAE-95 [11], *Streptomyces olivaceus* NEAE-119 [12] and *Streptomyces gulbargensis* [13]. The aim of the present work is the purification, characterization and immunogenicity assessment of an extracellular L-asparaginase produced under submerged fermentation from *Streptomyces brollosae* NEAE-115.

## Methods

### Microorganisms and cultural conditions

Soil samples collected from different areas in Egypt “Mansoura city, Damietta city, north western coast of Egypt from New Borg El-Arab to El Saloum, Janaklis (Beheira) and Brollos Lake” and three soil samples collected from Taif in Saudi Arabia were used for the isolation of actinomycetes, mainly *Streptomyces* spp*. Streptomyces brollosae* NEAE-115 was isolated from soil sample collected from Brollos Lake at the Mediterranean coast of Egypt. *Streptomyces* spp*.* had been isolated from the soil using standard dilution plate method on starch nitrate agar medium containing the following constituents (g/L): Starch, 20; CaCO_3_, 3; MgSO_4_.7H_2_O, 0.5; KNO_3_, 2; NaCl, 0.5; K_2_HPO_4_, 1; FeSO_4_.7H_2_O, 0.01; agar, 20. The incubation was carried at 30 °C for a period of 7 days. The obtained isolates of *Streptomyces* were purified and stored as spore suspensions in 20% (*v*/v) glycerol at − 20 °C for subsequent investigation.

### Screening of L-asparaginase production by plate assay method

According to De Jong [14], an increase in the culture filtrates pH is accompanied by L-asparaginase production. All actinomycetes isolates were tested for their abilities to produce L-asparaginase using plates containing asparagine dextrose salts agar (ADS agar) medium [15]. The constituents of the ADS agar medium were (%): L-asparagine 1.0; K_2_HPO_4_ 0.1; MgSO_4_ 0.05; dextrose 0.2; agar 1.5, pH was adjusted to 6.8–7. The medium was incorporated with pH indicator (phenol red, 0.009% w/v) prepared in ethanol according to the procedure of Gulati et al. [16]. The actinomycetes isolates were inoculated on ADS agar plates and incubated at 30 °C for 7 days. The formation of a pink zone around the microbial colonies due to change of pH was considered as a positive result for L-asparaginase production . Two control plates were prepared one was uninoculated medium while the other was without dye. The more potent isolate exhibiting L-asparaginase activity was selected for further study.

### Inoculum preparation

Three disks (8 mm each) were taken from old stock culture grown on starch nitrate agar medium for 7 days and inoculated into 50 mL of asparagine dextrose salts broth containing (%): L-asparagine 1.0; K_2_HPO_4_ 0.1; dextrose 0.2; MgSO_4_ 0.05. The inoculated 250 mL Erlenmeyer flasks were grown at 30 °C and 150 rpm for 48 h and cultured medium was further used for subsequent experiments.

### Production of L-asparaginase by submerged fermentation

The production of extracellular L-asparaginase from *Streptomyces brollosae* NEAE-115 was carried using the medium containing (g/L): Dextrose 2, starch 20, L-asparagine 10, KNO_3_ 1, K_2_HPO_4_ 1, MgSO_4_.7H_2_O 0.5, NaCl 0.1, pH 7. Fifty mL of the broth medium was dispensed in 250 mL Erlenmeyer conical flask. The inoculated flasks (with inoculum size of 4%, *v*/v) were incubated on a rotary shaker incubator at 150 rpm and 30 °C. After 7 days of incubation time, the broth was centrifuged with cooling centrifuge at 6000×*g* for 30 min at 4 °C and the clear supernatant served as crude enzyme.

### Assay of L-asparaginase activity

Extracellular L-asparaginase activity was determined according to the method of Wriston and Yellin [17] using nesslerization to measure the amount of liberated ammonia. The reaction mixture containing 1.5 mL of 0.04 M L-asparagine prepared in 0.05 M Tris-HCl buffer, pH 8.6 and 0.5 mL of the enzyme to make up the total volume to 2 mL. The tubes were incubated at 37 °C for 30 min. The reaction was terminated by the addition of 0.5 mL of 1.5 M trichloroacetic acid (TCA). The blank was run by adding TCA followed by enzyme preparation. To remove the precipitated protein, the reaction contents were centrifuged at 10,000×*g* for 5 min and the filtrate was collected. For quantification of liberated ammonia, 0.5 mL filtrate was diluted to 7 mL with distilled water and 1 mL Nessler’s reagent was added to the resulting mixture. The color reaction was allowed to proceed for 20 min before measuring the absorbance at 480 nm using Optizen Pop –UV/Vis spectrophotometer. A coloration of yellow indicates the presence of ammonia. However, a brown precipitate is formed at higher concentrations. The amount of liberated ammonia by the test sample was calculated by comparing the absorbance with a standard curve prepared from solutions of ammonium chloride as the ammonia source. One unit (U) of L-asparaginase is the amount of enzyme which generates 1μmole of ammonia in 1 min at 37 °C and pH 8.6.

### Assay of L-glutaminase

L-glutaminase activity was determined using L-glutamine as substrate according to Imada et al. [18] method and the released ammonia was measured by using Nesseler’s reagent.

### Purification of L-asparaginase from *Streptomyces brollosae* NEAE-115

The crude enzyme extract used in L-asparaginase purification was obtained from production medium centrifuged at 6000×*g* for 30 min and the resulted supernatant was transferred into a conical flask placed in ice cold condition with stirring. Finely powdered ammonium sulfate was added until complete dissolving takes place to give 45% saturation with ammonium sulphate and kept overnight in the refrigerator at 4 °C. The precipitate was collected by centrifugation at 11000×g for 30 min, while the supernatant was brought to 55–85% saturation with ammonium sulphate. Then, the formed precipitates were separately collected by centrifugation and dissolved in 50 mM Tris-HCl buffer pH 8.4. The dialysis of the ammonium sulphate was carried out in a pre-treated dialysis tube. Precipitate formed during dialysis was removed by centrifugation and was discarded. For the present study dialysis tubing (SERVA pro, 44,144, diameter 21 mm × 5 m) was used. After dialysis, the samples were used for protein estimation by the method of Lowry et al. [19] and enzyme was assayed by the direct nesslerization method according to the method of Wriston and Yellin [17] and stored at − 20 °C for further purification. The concentrated enzyme solution was applied to the ion exchange column of DEAE-Sepharose CL-6B (2.7 × 20 cm) that was pre-equilibrated with 50 mM Tris-HCl buffer (pH 8.4). It was eluted with the same buffer containing increased concentration (0.1–0.5 M) of NaCl solution to elute the enzyme at a flow rate of 10 mL per 1 h. Fractions of 2 mL were collected, after fractions dialysis, the samples were subjected to protein estimation [19] and assay of L-asparginase activity [17]. Fractions showing high L-asparaginase activity were collected for further use.

### Physicochemical characterization of the purified enzyme

The optimum pH of the purified enzyme was studied over the pH range of 4.5 to 10.5 with L-asparagine as a substrate dissolved in different buffers of 0.05 M: citric acid- Na_2_HPO_4_ (pH 4.5–7.5), Tris-HCl (pH 8.5) and glycine-NaOH (pH 9.5–10.5). The influence of temperature on L-asparaginase activity was analysed by incubating the assay mixture over the temperature range of 25 to 60 °C in 0.05 M Tris-HCl buffer under assay conditions. The optimum substrate concentration for the enzyme activity was determined by incubating the purified enzyme in the presence of different substrate concentration (1–10 mM). To evaluate the incubation time effect on L-asparginase activity, the reaction mixture was incubated for different times (10, 20, 30, 40, 50, 60, 70 and 80 min). Along the characterization of the enzyme, activity was determined as reported earlier.

### Determination of the kinetic parameters K_m_ and V_max_

The kinetic parameters, Michaelis–Menten constant (K_m_) and maximal velocity (V_max_) of the purified L-asparaginase were determined with different concentrations of L-asparagine (1–10 mM) as substrate and the data were fitted to a one-phase exponential association nonlinear regression curve using GraphPad Prism 5 software (GraphPad Software Inc., San Diego, CA). The enzyme activity was determined by measuring the rate of hydrolysis of L-asparagine under standard assay conditions using the Michaelis–Menten equation:

1 $$ {\mathrm{V}}_0=\frac{{\mathrm{V}}_{\mathrm{m}\mathrm{ax}}\left[\mathrm{S}\right]}{{\mathrm{K}}_{\mathrm{m}}+\left[\mathrm{S}\right]} $$

Whereas: initial reaction velocity (V_0_), substrate concentration [S], the Michaelis-Menten constant (K_m_) and maximal velocity (V_max_).

### Thermal stability of L-asparaginase

The stability of the L-asparaginase to temperature was determined by pre-incubating the reaction mixture (without its substrate) containing buffered enzyme for different time interval (0.0 to 90 min) with different temperatures (40, 50, 60, 70 and 80°С). After the end of the incubation periods, the enzyme was cooled and the residual activities were assayed.

### Estimation of deactivation rate constant (k_d_) and half-life time (T_1/2_)

The heat inactivation half-life (T_1/2_) of the enzyme and thermal deactivation constant (k_d_) of the purified L-asparaginase produced by *Streptomyces brollosae* NEAE-115 were determined by using GraphPad Prism 5 software (GraphPad Software Inc., San Diego, CA). It was assumed that enzyme thermal deactivation is a reaction follows the first order kinetics (single step two-stage theory) [20] as follows:

2 $$ \mathrm{E}\to {\mathrm{E}}_{\mathrm{d}} $$

Where, E is the active enzyme state and E_d_ is the deactivated state. The first order deactivation can be represented as follows:

3 $$ \frac{\mathrm{d}\mathrm{E}}{\mathrm{d}\mathrm{t}}=\kern0.5em -{\mathrm{K}}_{\mathrm{d}}\left[\mathrm{E}\right] $$

Where, K_d_ is the deactivation rate constant, [E] is concentration of the active enzyme.

Eq. (3) integrated with the initial condition, gives:

4 $$ \ln \left(\frac{{\mathrm{E}}_{\mathrm{d}}}{\mathrm{E}}\right)=\kern0.5em -{\mathrm{K}}_{\mathrm{d}}\mathrm{t} $$

This result is the exponential decay model. Therefore, by plotting log of (E_d_/E) against time, the deactivation rate constant value (K_d_) is obtained [21].

The half-life of the enzyme activity (T_1/2_) is the time it takes for the activity to reduce to a half of the initial activity value. The half-life time was determined by using the following equation:

5 $$ {\mathrm{T}}_{\raisebox{1ex}{$1$}\!\left/ \!\raisebox{-1ex}{$2$}\right.}\kern0.5em =\kern0.5em \frac{\ln \kern0.5em 2}{{\mathrm{k}}_{\mathrm{d}}}\kern0.5em =\kern0.5em \frac{0.693}{{\mathrm{k}}_{\mathrm{d}}} $$

Where: K_d_ is deactivation rate constant.

The deactivation rate constant (K_d_) at each temperature was determined from Arrhenius equation as:

6 $$ \ln \left(\frac{{\mathrm{k}}_{\mathrm{d}}}{{\mathrm{k}}_0}\right)=\kern0.5em -\left(\frac{\mathrm{E}}{\mathrm{RT}}\right) $$

Plotting the log of K_d_ as a function of the inverse of the absolute temperature. The values of the deactivation energy (E) and k_0_ (frequency factor, min^− 1^) were obtained from the slope and intercept of the plot of ln (k_d_) versus (1/T); respectively. R is the universal gas constant and T is absolute temperature.

### Effect of pH on L-asparaginase stability

To study the optimum pH for L-asparaginase stability, the enzyme in absence of its substrate was pre-incubated at room temperature for 0, 6, 12, 18, and 24 h in buffers of various pH values (pH 4.5–10.5). The residual activity was assayed under the standard assay conditions.

### Effect of metal ions, inhibitors and surfactants on L-asparaginase activity

The effects of various metal ions, inhibitors and surfactants on L-asparaginase activity were determined by preincubating the enzyme with individual metal ions solutions prior to adding the substrate at a final concentration of 5 mM concentration and inhibitors and surfactants at 1 mM concentration for 30 min [22] at 4 °C. The residual activity of the enzyme was measured under the standard assay conditions. The relative activities were determined by considering 100% activity of the enzyme without the addition of metal ions or inhibitors or surfactants as control.

### Molecular weight determination

The purity degree and the mass of the purified L-asparaginase enzyme was determined by sodium dodecyl sulfate polyacrylamide gel electrophoresis (SDS-PAGE) according to the method of Laemmli [23] with a 10% separating acrylamide gel (pH 8.8) and a 5% stacking gel (pH 6.8) containing 0.1% SDS. Gels were stained with coomassie brilliant blue R-250 followed by distaining step with a mixture of methanol- acetic acid and water in the ratio of 4:1:5. Molecular weight of L-asparaginase was determined using standard molecular weight protein marker ranged from 9 to 178 kDa.

### Experimental animals

Female Swiss albino mice with body weight of 20–30 g were received from Urology and Nephrology Center of Mansoura University, Mansoura, Egypt, used to compare the cytotoxic effect of L-asparaginase purified from *Streptomyces brollosae* NEAE-115 and those commercially available L-asparaginase from *E. coli* (Sigma-Aldrich, Product Number: A3809; CAS Number: 9015–68-3). Animals were housed in a conditioned atmosphere at temperature of 24 ± 1 °C and 55 ± 5% relative humidity with regular 12 h light/12 h dark cycles and free access to standard laboratory food and water. All experiments have been conducted under the regulations and the ethical guidelines for laboratory animals approved by the Ethical Committee of Faculty of Pharmacy, Mansoura University, Egypt.

### Transplantation of tumor

Ehrlich ascites carcinoma cells (EAC) were supplied by the Netherlands Cancer Institute. The cells were maintained in vivo in female Swiss albino mice by serial intraperitoneal transplantation [24] in the laboratory of Faculty of Pharmacy, Mansoura University, Egypt.

### In vivo experiment and evaluation of antitumor activity

The 7–10 days old EAC cells were used along experiment. Ascites fluid from tumor-bearing mice was withdrawn using a needle aspiration under aseptic conditions from the peritoneal cavity, and subjected to three times washing with normal saline followed by centrifugation at 67×*g*. Tryphan blue exclusion test was used for tumor viability determination and haemocytometer was used for cells counting. To get ascitic fluid with a concentration of 5 × 10^5^ viable EAC cells/0.1 mL of tumor cell suspension normal saline was used and was injected into the right thigh of the lower limb of mice to obtain ascitic tumor [25].

The largest tumor diameter and its perpendicular were measured by a digital caliper and used for tumor growth determination [26].

7 $$ \mathrm{Tumor}\ \mathrm{size}\ \left({\mathrm{mm}}^3\right)=0.5\ \mathrm{Xa}\ {\mathrm{Xb}}^2 $$

Whereas: largest tumor diameter (a) and its perpendicular (b).

When the primary tumor reached a size of 50–100 mm^3^, Swiss albino mice were divided into three groups of six each. Group (1) received normal saline (EAC-bearing control, 5 mL/kg). Group (2) received commercial L-asparaginase. Group (3) received L-asparaginase produced by *Streptomyces brollosae* NEAE-115. L-asparaginase treatments were given five days after the inoculation, two times a week for two weeks. After 24 h of the last dose and then 18 h of fasting, animals of each group were sacrificed by cervical dislocation to determine the antitumor activity of the tested L-asparaginase. Antitumor activity was calculated by the determination of ΔT (change of tumor size in the treatment group) and ΔC (change of tumor size in the control). The degree of tumor growth inhibition can be obtained from ΔT/ΔC X 100 [26].

### Immunogenicity assessment

Two groups each of 6 mice received intraperitoneal administration of either commercial L-asparaginase (250 U/kg) or *Streptomyces brollosae* NEAE-115 L-asparaginase (250 U/kg) twice a week for 4 weeks. Levels of specific antibodies (IgG immunoglobulin) against L-asparaginase or commercial product were measured in sera using ELISA method and microplate reader was used at absorbance of 450 nm. The direct enzyme-linked immunosorbent assay (ELISA) was carried out to evaluate the presence of asparaginase–specific IgG antibodies in serum samples by employing horseradish peroxidase–conjugated goat-anti mouse IgG (from Southern Biotech). All ELISA steps were conducted at room temperature, and 2.5% casein (w/v) was utilized as binding and blocking buffer. The assay steps and conditions were as follows: 2 h blocking of asparaginase-coated plates followed by extensive washing using PBS-Tween, incubated with diluted serum samples for 2 h, washed, incubated with detection antibody for 1 h, washed, developed with 3, 3′, 5, 5′-tetramethylbenzidine substrate for 30 min, and quenched with 9.8% (*v*/v) H_2_SO_4_ in water. IgG titers were calculated.

## Results

Production of the L-asparaginase by *Streptomyces brollosae* NEAE-115 was detected by plate assay. The enzyme production was indicated by color change in the medium from yellow to pink zone surrounding the colony. A broad diameter of the pink zone around the colony indicated that the organism was an efficient producer of L-asparaginase. During shake flasks production of L-asparaginase, the mycelial growth has been formed as large, spherical pellets which may lead to better yield of L-asparaginase than growth as free filaments. The glutaminase activity of L-asparaginase was investigated and the enzyme is free from glutaminase activity.

### Purification of L-asparaginase from *Streptomyces brollosae* NEAE-115

**Fig. 1:**
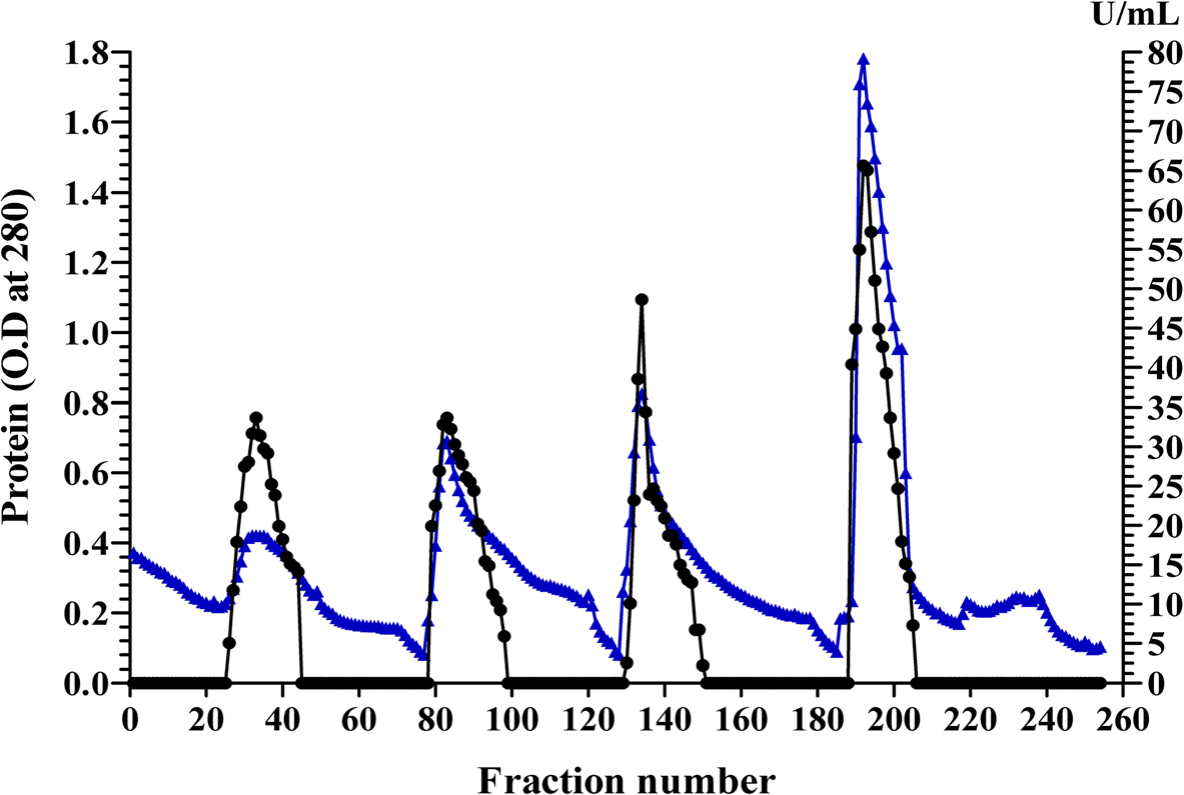
Purification of L-asparaginase produced by *Streptomyces brollosae* NEAE-115 using ion exchange on DEAE Sepharose. () refer to protein, (●) refer to L-asparaginase activity

Purification of L-asparaginase of *Streptomyces brollosae* NEAE-115 was carried out using crude culture filtrate having a total activity of 30538.87 U, protein content 3120.42 mg and specific activity of 9.786 U/mg protein. The dialysed ammonium sulphate concentrated enzyme had a specific activity of 69.004 U/mg protein with protein content of 158.86 mg and the enzyme recovery was 35.895% with purification fold of 7.051 (Table 1). The dialyzed enzyme obtained after ammonium sulphate precipitation was used to purify the enzyme with DEAE Sepharose CL-6B packed column which resulting 260 fractions with major L-asparaginase peak on the chromatogram (Fig. 1). The final purification step, DEAE Sepharose CL-6B column, showed a total enzyme activity of 795.152, protein content of 10.371 mg with enzyme specific activity of 76.671 U/mg of protein and the results of all purification steps applied to purify L-asparaginase produced by *Streptomyces brollosae* NEAE-115 are summarized in Table 1.

**Table 1:** Summary of the purification steps of the L-asparaginase produced by *Streptomyces brollosae* NEAE-115

Purification step	Total protein content (mg)	L-asparaginase activity		Recovery (%)	Purification fold
		Total activity (U)	Specific activity (U/mg protein)		
Culture filtrate	3120.42	30538.87	9.786	100	1
(NH_4_)_2_SO_4_, post dialysis	158.86	10,962	69.004	35.895	7.051
Ion exchange on DEAE Sepharose	10.371	795.152	76.671	7.254	7.835

### SDS-PAGE and molecular weight determination

**Fig. 2:**
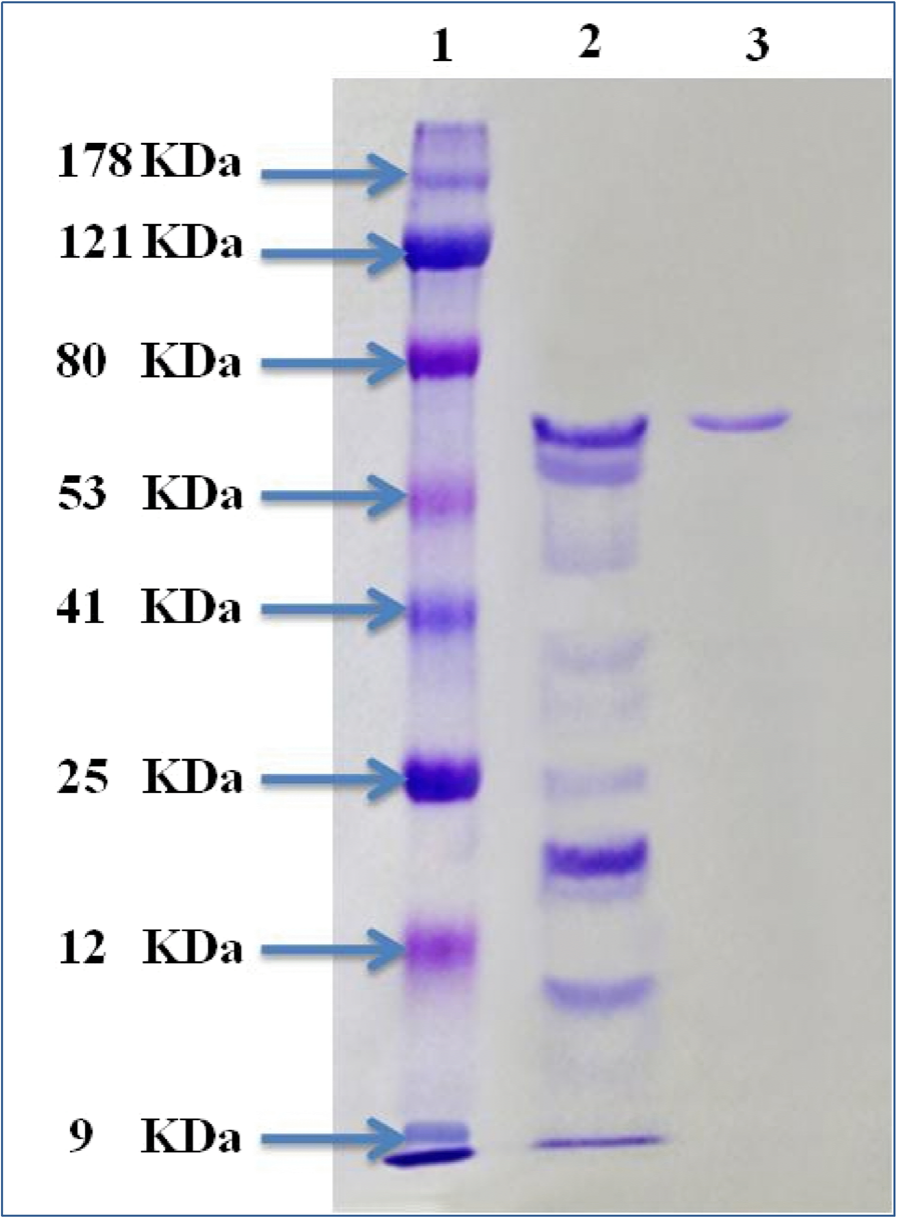
SDS-polyacrylamide gel electrophoresis of the purified L-asparaginase from *Streptomyces brollosae* NEAE-115. Lane 1: Protein marker; Lane 2: Ammonium sulphate fraction; Lane 3: Purified protein

The purity and molecular weight of the purified enzyme was determined by SDS-PAGE according to the method of Laemmli [23] with gel system contained a separating gel of 10% and a stacking gel 5%. After the electrophoresis, protein bands were visualized by staining with coomassie brilliant blue R-250. The resolved electrophoretic bands of the enzyme from different purification steps on the SDS-PAGE showed that the enzyme purity was successfully improved. SDS–PAGE separation of the enzyme preparation showed no detectable contamination and electrophoretic resolved only one distinctive band. By using different molecular markers with known molecular weight ranged from 9 to 178 kDa, the molecular mass of the purified L-asparaginase was estimated. The relative mobility (R_f_) value was calculated for the distinctive single band. The calculated R_f_ value is compared with the different standard proteins of known molecular weight. Hence the molecular weight for individual band is determined with a molecular mass of 67 kDa (Fig. 2).

### Physicochemical properties of the purified L-asparaginase

The purified L-asparginase of *Streptomyces brollosae* NEAE-115 was characterized for its activity at different pH levels, temperatures, substrate concentrations and incubation times.

### Effect of pH on L-asparaginase activity

**Fig. 3:**
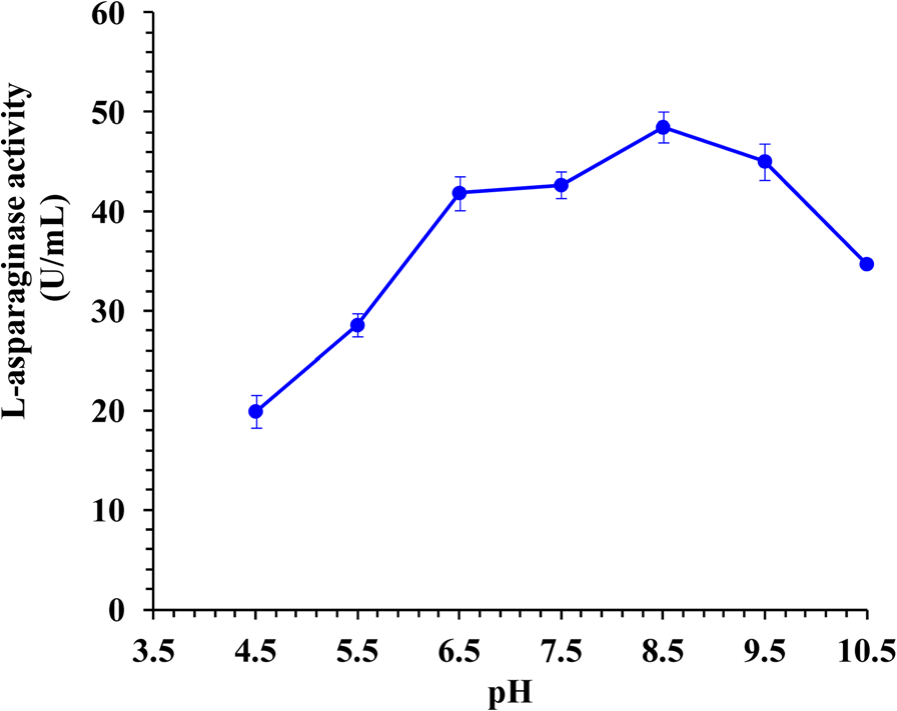
Activity of L-asparaginase as a function of the pH of the reaction

The pH of the reaction played a vital role in most of enzymatic processes. There are number of reports on the enzyme activity at near physiological range. Fig. 3 shows that the purified L-asparaginase was active over a broad pH range of 4.5 to 10.5 with an optimum of 48.462 U/mL at pH 8.5. At higher pH’s, the enzyme activity was decreased. The enzyme retains 71.595% activity even at pH 10.5 and 58.919% at pH 5.5.

### Incubation time effect on enzyme activity

**Fig. 4:**
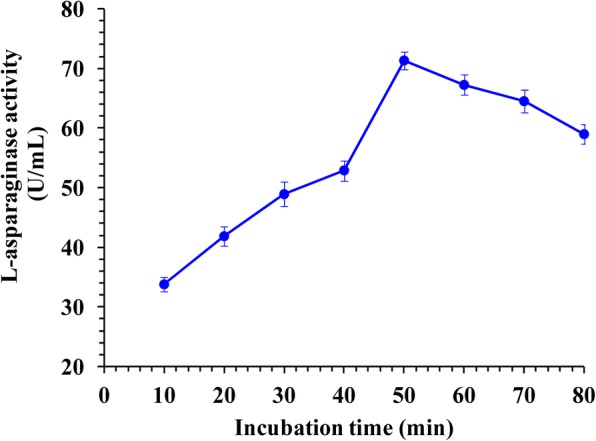
Effect of different incubation time on L-asparaginase activity

L-asparaginase activity of *Streptomyces brollosae* NEAE-115 (Fig. 4) was gradually increased with increasing incubation time up to 50 min (L-asparaginase activity of 71.327 U/mL). After which only a slight decrease in L-asparaginase activity was observed.

### Temperature effect on L-asparaginase activity

**Fig. 5:**
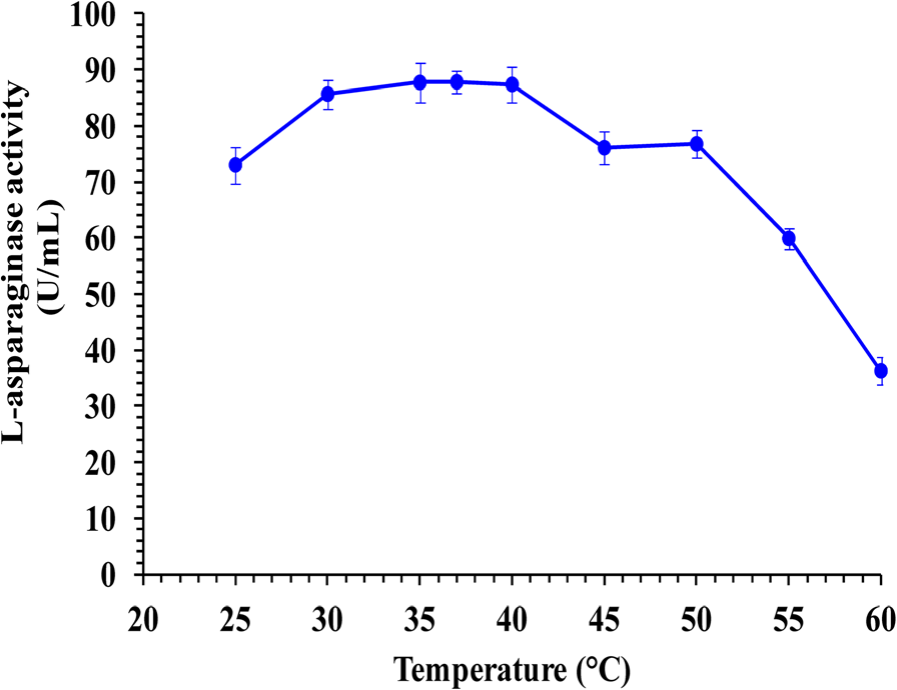
Effect of the temperature on L-asparaginase activity

Figure 5 shows that the purified L-asparaginase was active over a wide range of temperature range of 25 to 60 °C with an optimum L-asparaginase activity of 87.81 U/mL at 37 °C and lower L-asparaginase activity observed at higher temperatures.

### Effect of substrate concentration on the activity of L-asparaginase

**Fig. 6:**
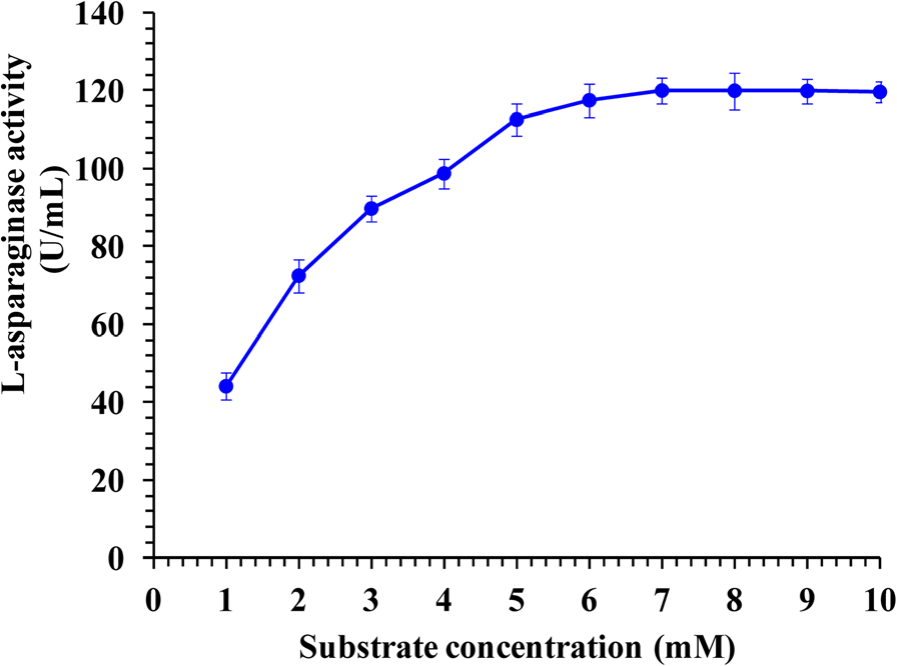
Effect of the substrate concentration of the reaction on L-asparaginase activity

**Fig. 7:**
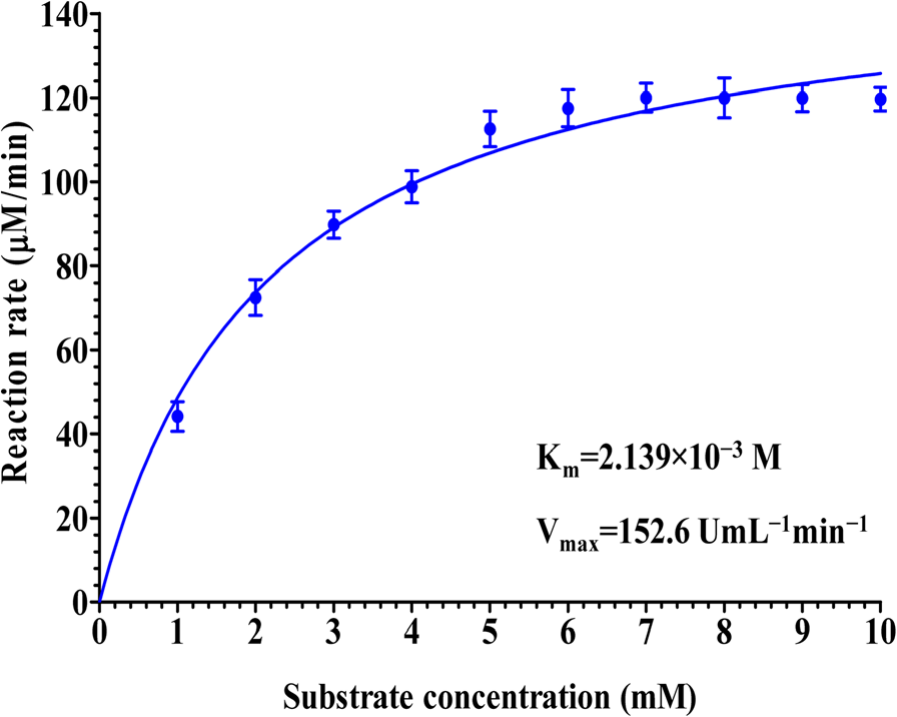
Michaelis-Menten plot for L-asparaginase produced by *Streptomyces brollosae* NEAE-115

The results in Fig. 6 showed the effect of various substrate concentrations ranging from 1 to 10 mM on the activity of L-asparaginase and verified the optimum required substrate concentration that gives the maximum L-asparaginase activity. The obtained results illustrated a gradual enzyme activity increase with increasing of the substrate concentration up to 7 mM demonstrating the optimum substrate concentration for enzyme activity. However, higher substrate concentration (8 to 10 mM) resulted in decreasing of enzyme activity. Activation of asparagine hydrolysis was investigated in terms of change in values of kinetic constants (K_m_ and V_max_). The K_m_ and V_max_ values were calculated through Michaelis-Menten plot by plotting the relation between different substrate concentrations [S] versus enzyme activity (V), using enzyme kinetic template of Graph-Pad Prism 4 software. Michaelis-Menten plot showed in Fig. 7 illustrated the K_m_ and V_max_ values for L-asparaginase enzyme. The plot gave K_m_ value of 2.139 × 10^− 3^ M and V_max_ of 152.6 UmL^− 1^ min^− 1^ for the hydrolysis of L-asparagine by L-asparaginase from *Streptomyces brollosae* NEAE-115 and K_m_ value of 1.76 × 10^− 4^ M and V_max_ of 121.7 UmL^− 1^ min^− 1^ for the hydrolysis of L-asparagine by commercially available L-asparaginase from *E. coli* (Sigma-Aldrich, Product Number: A3809; CAS Number: 9015–68-3)*.*

### Thermal stability

**Fig. 8:**
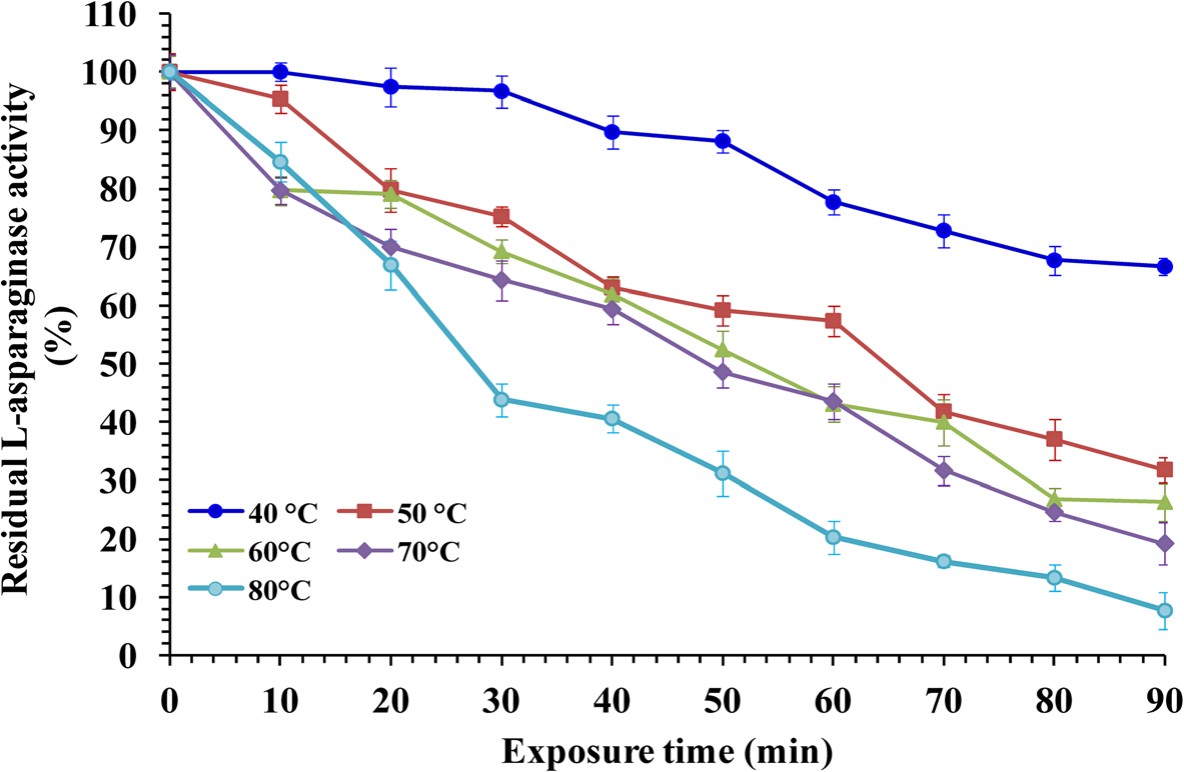
Thermal stability of L-asparaginase as a function of the time of the reaction

The temperature impact on L-asparaginase stability was studied and the maximum L-asparaginase stability was recorded at 40 °C (Fig. 8) with retained enzyme activity of 97.44% from initial activity after incubation time of 20 min. However, enzyme exposure to higher temperature and longer incubation time of 50 °C and 80 °C for 90 min led to observed rapid decrease in L-asparaginase activity with residual activity of 31.84 and 7.69%; respectively.

On the other hand, to reveal heat inactivation half-life time (T_1/2_) of the enzyme at the used temperatures and heat deactivation constant (k_d_), the linear regression of the obtained results was performed using Graph-Pad Prism software by fitting the data points to first order equation according to Eqs. (4) and (5) and their values were listed in Table 2. The stability of L-asparaginase was expressed as a percentage of residual activity compared to the initial activity of the un-treated enzyme activity which considered as a control (100%). The enzyme half life time (T_1/2_) of L-asparaginase was found to be 65.02 min at 50°С and 62.65 min at 60°С. However, enzyme activity destruction was recorded at 80 °C with low half-life time (49.60 min).

**Table 2:** Half life time (T_1/2_) and heat deactivation constant (k_d_) of L-asparaginase produced by *Streptomyces brollosae* NEAE-115

Temperature (°C)	Half life time (min)	k_d_ (min)	*R*^*2*^ value
40	116.50	0.006	0.94
50	65.02	0.011	0.98
60	62.65	0.011	0.97
70	59.91	0.012	0.97
80	49.60	0.014	0.93

### pH stability

**Fig. 9:**
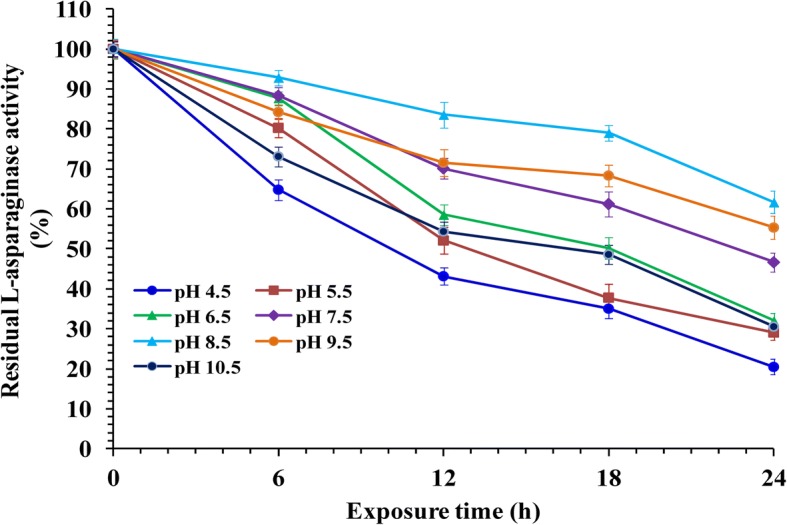
pH stability of L-asparaginase at different times of the reaction

The purified L-asparaginase was more stable in alkaline pH than the acidic one; it retains 92.81% activity at pH 8.5 even after incubation for 6 h and 61.64% activity after 24 h (Fig. 9). Moreover, the enzyme retains about 84.22% of its activity after 6 h at the pH 9.5. At pH 4.5 the enzyme retained 64.80% activity after 6 h and the residual activity was 20.47% after 24 h. Half life time values in hours (T_1/2_) based on pH studies of L-asparaginase produced by *Streptomyces brollosae* NEAE-115 are represented in Table 3. The enzyme half life time of L-asparaginase was found to be 33.135 h at pH 8.5 and 28.506 h at pH 9.5. However, enzyme activity destruction was recorded at pH 4.5 with low half-life time (15.888 h).

**Table 3:** Half life time values in hours (T_1/2_) based on pH studies of L-asparaginase produced by *Streptomyces brollosae* NEAE-115

pH	T_1/2_ (h)	*P*-value	*R*^*2*^ value
4.5	15.888	0.0077	0.9321
5.5	16.276	0.0029	0.9644
6.5	17.289	0.0017	0.9748
7.5	22.422	0.0003	0.9921
8.5	33.135	0.0039	0.9563
9.5	28.506	0.003	0.9634
10.5	18.355	0.0039	0.9564

### Effect of metal ions, inhibitors and surfactants on L-asparaginase activity

**Fig. 10:**
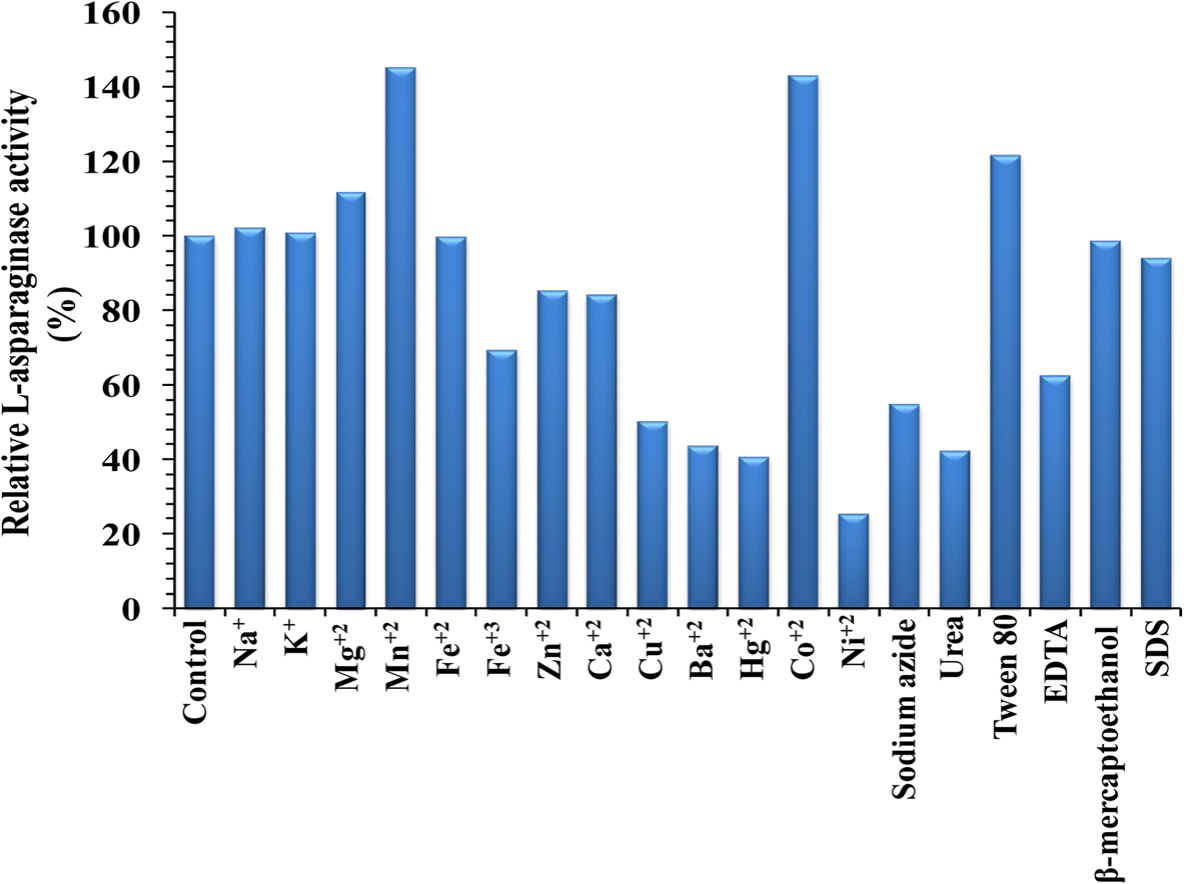
Effect of metal ions, inhibitors and surfactants on L-asparaginase activity

The effect of various metal ions, inhibitors and surfactants on the enzyme activity is represented in Fig. 10. The purified L-asparaginase from *Streptomyces brollosae* NEAE-115 was not significantly affected by the presence of metal ions (Na^+^, K^+^, Fe^2+^) and β-mercaptoethanol. It is evident from the Fig. 10 that the enzyme activity enhanced considerably in the presence of Mg^2+^ (111.81%) and was maximal in the presence of Mn^2+^, Co^2+^ and Tween 80 by 145.15, 143.04 and 121.52%; respectively (acted as activators for L-asparaginase activity). However, a slight decrease, around 14.8 and 16%, in L-asparaginase activity was observed in the presence of Zn^2+^ and Ca^2+^; respectively. Moreover, the presence of EDTA and urea acted as inhibitors of L-asparaginase activity reducing its activity by 37.55 and 57.8%; respectively. The presence of Ni^2+^, Hg^2+^, Ba^2+^ and Cu^2+^ acted as a potent inhibitor, reducing about 74.68, 59.5, 56.4 and 49.8%; respectively of the L-asparaginase activity. The presence of sodium azide also acted as an inhibitor, reducing about 45.15% of the L-asparaginase activity.

### Antitumor activity of purified L-asparaginase and immunogenicity assessment

**Fig. 11:**
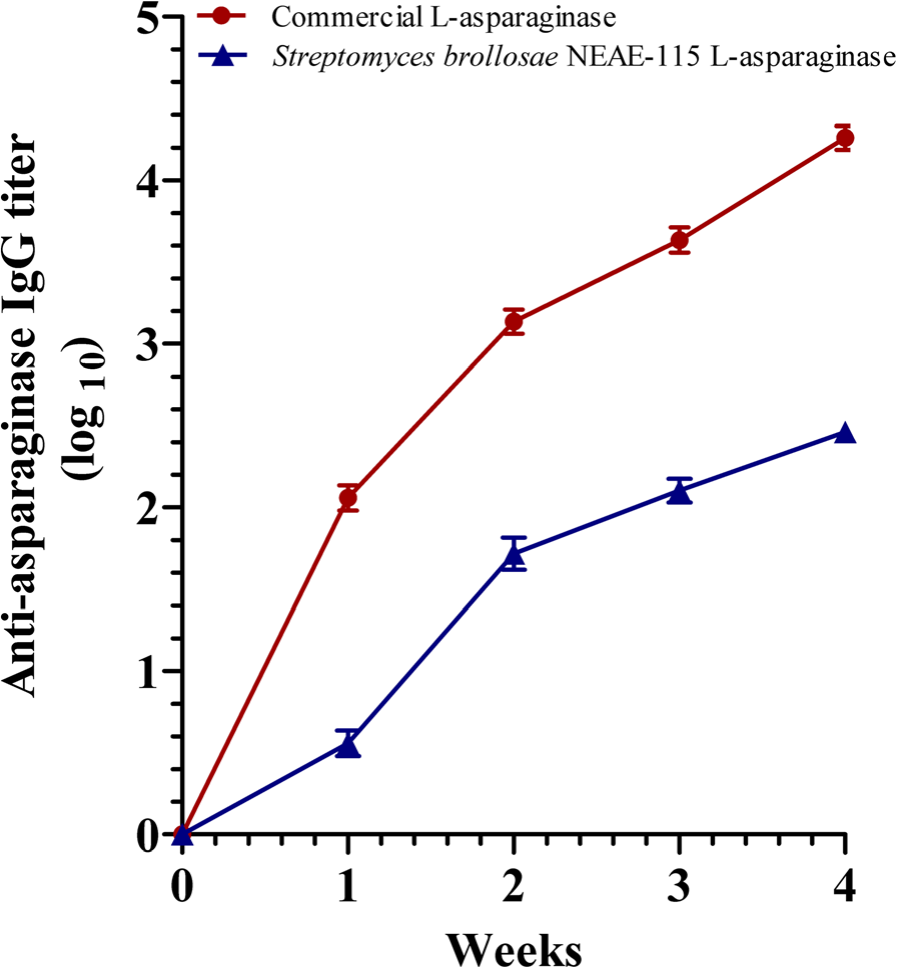
Time course of anti-asparaginase IgG development in serum of mice receiving commercial L-asparaginase or *Streptomyces brollosae* NEAE-115 L-asparaginase, represented as log_10_ titer. Mann-Whitney U test, *P < 0.05

Treatment with commercial L-asparaginase significantly decreased the relative tumor size compared to the control group, showing 33% tumor growth (i.e. 67% tumor growth inhibition). While mice treated with *Streptomyces brollosae* NEAE-115 L-asparaginase showed 79% tumor growth inhibition showing a significantly higher cytotoxic effect when compared to commercial L-asparaginase group (Table 4). One of the most important limitation of L-asparaginase as anticancer drug is that the allergic reactions exhibited by immune system of the patients receiving the medication of L-asparaginase. Characterization of the immunogenicity profile of L-asparaginase in mice and the levels of specific antibodies (IgG immunoglobulin) against L-asparaginase was measured in sera using ELISA method. Therapeutic efficacy and evaluation of anti-L-asparaginase IgG antibody concentrations in mice receiving *Streptomyces brollosae* NEAE-115 L-asparaginase preparations was carried out simultaneously with the commercial L-asparaginase in order to evaluate the superiority of the new product. There was a gradual increase in L-asparaginase IgG titre in both commercial L-asparaginase or *Streptomyces brollosae* NEAE-115 L-asparaginase-inoculated mice (Fig. 11). However significant anti-asparaginase IgG titer was observed in commercial L-asparaginase group when compared to the other group of mice inoculated by *Streptomyces brollosae* NEAE-115 L-asparaginase due to the highly immunogenic nature of the commercial asparaginase. The results demonstrated that the immunogenicity of the *Streptomyces brollosae* NEAE-115 L-asparaginase was remarkably reduced.

**Table 4:** Effect of L-asparaginase on tumor size in mice after treatment

Treatment	Tumor size (mm^3^)			
	Day 0	Day 14	ΔT/ΔC (%)	% Inhibition
Control	69.0 ± 2.2	585.0 ± 40.8	100	0
Commercial L-asparaginase	67.0 ± 4.5	240.7 ± 9.4*	33	67
*Streptomyces brollosae* NEAE-115 L-asparaginase	77.6 ± 3.7	188.3 ± 8.33$	21	79

Data are expressed as mean ± SEM, *n* = 6

* Significantly different from control group using One-Way ANOVA followed by Tukey-Kramer multiple comparisons test (*P* < 0.05). $ Significantly different from commercial L-asparaginase-treated group using One-Way ANOVA followed by Tukey-Kramer multiple comparisons test (*P* < 0.05). Mann-Whitney U test, *P < 0.05

## Discussion

In the current study, L-asparaginase produced by *Streptomyces brollosae* NEAE-115 was detected by forming pink zone surrounding the colony. The pink zone resulted from the breakdown of amide bond in asparagine in the growth medium by L-asparaginase to aspartate and ammonia that changed the color of phenol red to pink [27].

The produced enzyme was partially purified by ammonium sulphate precipitation and pure enzyme was reached using ion-exchange chromatography. Recently L-asparaginase enzyme from strains *Streptomyces noursei* [28] and *Streptomyces gulbergenesis* [13] with an overall purification of 98.23 and 82.12 fold in final purification were carried out successfully. Sahu et al. [27] extracted the L-asparaginase from the actinomycete strain LA-29 isolated from the gut contents of the fish and the enzyme was purified 18-fold and from which 1.9% of protein was recovered and showed the specific activity of about 13.57 IU/mg of protein. Narayana et al. [29] and El-Bessoumy et al. [30] reported 99.3 and 106 fold purification of L-asparaginase from *Streptomyces albidoflavus* and *Pseudomonas aeroginosa* 50,071; respectively, by CM Sephedex C-50 column chromatography.

SDS–PAGE separation revealed only one distinctive band of the enzyme preparation, no detectable contamination. L-asparaginase was therefore a homogenous protein. The molecular weight for the individual band is determined with a molecular mass of 67 kDa. The molecular weight of the L-asparaginase was found to be varied according to the source of enzyme. Purified L-asparaginase from *Bacillus* sp. [31], *Streptomyces gulbargensis* [13], *Streptomyces albidoflavus* [29], *Streptomyces* PDK2 [32], and *Streptomyces noursei* [28] exhibited a molecular weight of 45, 85, 112, 140 kDa and 102 kDa; respectively.

The amidases enzymes such as L-asparaginase are generally stable and active at neutral and alkaline pH, whereas, earlier reporters showed optimum amidase activity at pH ranging from 5.0 to 9.0 [33]. The optimum pH 8.5 was also reported for L-asparaginase purified from *Streptomyces* sp. PDK7 [32]. However, the optimal L-asparaginase activity from *Streptomyces gulbargensis* was reported at pH 9.0 [13]. Narayana et al. [29] have reported the maximum L-asparaginase at pH 7.5 by *Streptomyces albidoflavus*. Khamna et al. [34] have also reported maximum L-asparaginase activity at pH 7.0 using *Streptomyces* sp.

The L-asparaginase activity of *Streptomyces brollosae* NEAE-115 was gradually increased with increasing incubation time up to 50 min. However, longer time of incubation with substrate resulted in reduction of L-asparaginase activity which may due to the product inhibition. The purified L-asparaginase from *Streptomyces noursei* showed maximum activity at 35 min of incubation time [28]. In addition, L-asparaginase purified from *Pseudomonas aeruginosa* 50,071 reached its maximum activity at 30 min [30].

L-asparaginase was active over a wide range of temperature from 25 to 60 °C with an optimum L-asparaginase activity of 87.81 U/mL at pH 37 °C and lower L-asparaginase activities observed at higher temperatures. Similarly, Siddalingeshwara and Lingappa [35] recorded optimum temperature 37 °C for maximum enzyme activity from *Aspergillus terreus* KLS2. This property of enzyme makes it most suitable for complete elimination of asparagine from the body when tumor patient treated with L-asparaginase. Comparable results were reported for the maximum activity of purified L-asparaginase from *Streptomyces gulbargensis* at 40 °C [13].

Michaelis-Menten plot gave a K_m_ value of 2.139 × 10^− 3^ M with L-asparagine as substrate and V_max_ of 152.6 UmL^− 1^ min^− 1^ for the hydrolysis of L-asparagine by L-asparaginase from *Streptomyces brollosae* NEAE-115 and K_m_ value of 1.76 × 10^− 4^ M and V_max_ of 121.7 UmL^− 1^ min^− 1^ for the hydrolysis of L-asparagine by commercially available L-asparaginase from *E. coli* (Sigma-Aldrich, Product Number: A3809; CAS Number: 9015–68-3)*.* Senthil Kumar and Selvam [36] have reported the apparent K_m_ and V_max_ of 5.98 × 10^− 2^ M and 3.547 IU/μg; respectively in *Streptomyces radiopugnans* MS. Table 5 summerize the biochemical properties of some microbial L-asparaginases [13, 21, 22, 29, 30, 36–48].

**Table 5:** Biochemical properties of some microbial L-asparaginases

Microbial source	pH optima	Temperature optima (°C)	K_m_ (M)	V_max_	Specific activity (U/mg protein)	Molecular weight (kDa)	References
*Streptomyces albidoflavus*	7.5	35	–	–	437	112	[29]
*Streptomyces gulbargensis*	9	40	–	–	2053	85	[13]
*Streptomyces radiopugnans* MS	6	40	5.98 × 10^−2^	3.547 IU/μg	5035.28	133.25	[36]
*Enterobacter cloacae*	7–8	35–40	1.58 × 10^−3^	2.22 IU/μg	105.07	52	[37]
*Pseudomonas aeruginosa*	9	37	0.147 × 10^− 3^	35.7 IU	1900	160	[30]
*Vibrio cholerae*	7	37	1.1 × 10^− 3^	1006 μM/min	648.9	132	[21]
*Pseudomonas stutzeri*	9	37	1.45 × 10^−4^	–	732.3	34	[38]
*Pseudomonas fluorescens*	8–9		4.1 × 10^− 4^	–	500	70	[39]
*Azotobacter vinelandii*	8.6	48	1.1 × 10^− 4^		2.47	84	[40]
*E. coli*	7–8	37	3.5 × 10^− 3^	–	150–250	139	[41–43]
*Erwinia aroideae*	8.2	45	2.8 × 10^− 3^	–	–	155	[44]
*Corynebacterium glutamicum*	7	40	2.5 × 10^− 3^	–	2020	80	[45]
*Bacillus coagulans*	8.8–9.7	55	5 × 10^− 3^	5.83 × 10^− 5^ M/h	10.9	84	[46]
*Bacillus licheniformis*	6–10	40	1.4 × 10^−5^	4.03 IU	697.09	134.8	[47]
*Aspergillus oryzae* CCT 3940	8	50	0.66 × 10^−3^	313 IU/mL	91	115	[48]
*Fusarium culmorum* ASP-87	8	45	3.57 × 10^−3^	0.5 μmol/mL/min	16.66	90	[22]

L-asparaginase of different microbial sources has different K_m_ value which defined as the half maximal velocity of the enzymatic reaction as a function of substrate concentrations and stating the filling of half of the enzyme active sites in the tested sample in the steady state. Therefore, K_m_ values illustrate the affinity of the enzyme for its substrate [49]. In consequence, lower and higher K_m_ values mean stronger binding ability and lower enzyme affinity to its substrate; respectively. However, the kinetic constants of the enzymes in terms of K_m_ and V_max_ can be affected by many factors, including, enzyme type, enzyme different forms (crude, modified or purified), enzyme source, used conditions (temperature, pH, etc.), substrate type and the used procedure of assay [50]. The rate of an enzyme-catalyzed reaction increases with the increasing of the substrate concentration beyond a certain level called maximal velocity rate (V_max_); at V_max_ increase in substrate concentration does not cause any increase in reaction rate as there is no more enzyme available for reacting with substrate. The maximal rate, V_max_, reveals the number of substrate molecules converted into product by the enzyme per unit of time when the catalytic sites on the enzyme are fully saturated with its substrate [51].

According to the data reported under this investigation, the maximum thermal stability behavior of L-asparaginase was at 40 °C. An earlier study demonstrated by Senthil Kumar and Selvam [36], the pre incubation at 40 °C for 60 min of the purified L-asparaginase from *Streptomyces radiopugnans* MS1 resulted in no significant loss of enzyme activity. Similar results were recorded with many other microorganisms such as *Streptomyces noursei* [28], *Pseudomonas stutzeri* MB 405 [38] and *E. carotovora* [52]. “Enzyme stability and thermal deactivation are considered the major constraints in rapid development of biotechnology process. In addition, it considered a very important tool used in enzyme selection for industrial uses”.

The purified L-asparaginase was more stable in alkaline pH and similar findings were reported for L-asparaginase extracted from *Pseudomonas stutzeri* MB–405 which is maximally stable at pH range from 7.5 to 9.5 [38] .

Among the different metal ions tested Mn^2+^ enhanced the L-asparaginase activity by 18% as reported by earlier workers on *Fusarium culmorum* ASP-87 and *Mucor hiemalis* [22, 53]. Husain et al. [37] reported that the monovalent cations such as Na^+^ and K^+^ enhanced the activity of *Enterobacter cloacae* L-asparaginase. K^+^ acted also as enhancer on *Pectobacterium carotovorum* asparaginase [54]. Radha et al. [21] reported that the activity of *Vibrio cholerae* L-asparaginase was enhanced in presence of Ca^2+^ to 130%. Also, Meghavarnam and Janakiraman [22] reported that the non ionic surfactant tween 80 was found to enhance the activity of the enzyme by 16%. Senthil Kumar and Selvam [36] reported that the EDTA acts as an inducer in *Streptomyces radiopugnans* MS1. EDTA as metal chelator agent had no effect on *P. carotovorum* asparaginase [54]. However, L-asparaginase was completely inhibited by EDTA as reported by Borkotaky and Bezbaruah [55]. Whereas, EDTA inhibited the activity of fungal L-asparaginase from *Trichoderma viride* by 88% [56]. Also, the L-asparaginase activity of *Fusarium Culmorum* ASP-87 was inhibited by EDTA, SDS and Cu^2+^ [22]. Husain et al. [37] reported that the divalent and trivalent cations, Ca^2+^, Mg^2+^, Zn^2+^, Mn^2+^, and Fe^3+^ inhibited the enzyme activity. Radha et al. [21] reported that the activity of *Vibrio cholerae* L-asparaginase was inhibited by divalent cations Ni^2+^, Mg^2+^, Fe^2+^, Mn^2+^, Zn^2+^ and the complete loss in the activity was perceived in the presence Cu^2+^. Whereas, Archana and Raja [57] reported that Cu^2+^ inhibited the activity of L-asparaginase produced by *Aspergillus nidulans* by 84%. Kumar et al. [54] reported that Hg^2+^ inhibited the L-asparaginase activity produced by *Pectobacterium carotovorum* by 80%. However, L-asparaginase was completely inhibited by Fe^2+^ and Ni^2+^ [58] and Cu^2+^ and Zn^2+^ [59]. While, Husain et al. [37] reported that the asparaginase activity did not detected when enzyme was incubated with metal ions viz. Cd^2+^, Ni^2+^ and Hg^2+^. Other metal ions like Ca^2+^ and Mg^2+^ did not have much effect on enzyme activity [22] and β-mercaptoethanol did not have any effect on fungal L-asparaginase activity from *Trichoderma viride* [56].

Mn^2+^, Co^2+^ and Mg^2+^ ions increase the enzyme activity suggests that these metals ions can serve as co-factors, which can help to activate the enzymatic reaction. Mg^2+^ was thought to be the activating metal; Mg^2+^ may activate the substrate, bound directly to the enzyme-substrate complex. Mg^2+^ locks the enzyme-substrate complex in place and then rapidly causes release of the reaction products [60]. This corresponds to fast dissociation rates for the enzyme-product complex rendering more favorable substrate binding sites. Metal ions play a crucial role in maintaining the active configuration of the enzymes at elevated temperatures by protecting them against thermal denaturation [61]. It was reported that the divalent cations Mn^2+^ and Mg^2+^ increased the thermal stability of *Bacillus* alkaline proteases [62]. Whereas, the monovalent cations, Na^+^ were found to enhanced asparaginase activity, indicates that the enzyme might contain Na^+^ ions [37]. Thakur et al. [53] found that Mn^2+^ enhanced L-asparaginase activity and concluded that L-asparaginase is in general a metal-activated enzyme. Tween 80 was found to enhance the activity of the enzyme by 21.52%. Surfactants appear to increase quick and direct contact of enzymes with substrate sites [63]. McAllister et al. [64] reported that Tween 80 increased the stability and substrate binding capacity of enzymes under in vitro conditions.

One of the most important limitation of L-asparaginase as anticancer drug is that the allergic reactions exhibited by immune system of the patients receiving the medication of L-asparaginase. The patient immune system reacts in many different ways against the drug such as, the development of high titers of serum IgG antibodies which in the majority of cases interfere with the therapeutic effect of the enzyme [65]. Drug’s immunogenicity is fundamental obstacle which limits the therapy with foreign proteins in humans. A real immunological tolerance that would require antigen specific T-cell mediated immunosuppression is difficult to achieve. One way to overcome this problem for a limited time is to switch to another preparation [66].

## Conclusions

This study is clearly revealed that soils can be considered as a rich source of L-asparaginase producing actinomycetes. *Streptomyces brollosae* NEAE-115 isolated from Egypt soil has the ability to produce a significant amount of L-asparaginase. A pure and efficient enzyme activity of L-asparaginase could be obtained using only two purification steps. The excellent characteristics of this enzyme like high catalytic activity over a wide range of temperature and pH, high substrate specificity, maximum activity at body temperature and its considerable thermal and pH stabilities, makes it highly valuable to be exploited as a potent anticancer agent. Furthermore, mice treated with *Streptomyces brollosae* NEAE-115 L-asparaginase showed 79% tumor growth inhibition with higher cytotoxic effect when compared to commercial L-asparaginase group. The current investigation concluded the isolated *Streptomyces brollosae* NEAE-115 used in this study could be a valuable potential source for L-asparaginase.

## Abbreviations

ELISA: Enzyme-linked immunosorbent assay
IgG: Immunoglobulin G
KDa: kilodalton
K_m_: Michaelis–Menten constant
SDS–PAGE: Sodium dodecyl sulfate–polyacrylamide gel electrophoresis
T_1/2_: Half-life time
V_max_: Maximal velocity
